# Piezo1 Mechanosensitive Ion Channel Mediates Stretch-Induced Nppb Expression in Adult Rat Cardiac Fibroblasts

**DOI:** 10.3390/cells10071745

**Published:** 2021-07-09

**Authors:** Meike C. Ploeg, Chantal Munts, Frits W. Prinzen, Neil A. Turner, Marc van Bilsen, Frans A. van Nieuwenhoven

**Affiliations:** 1 Department of Physiology, Cardiovascular Research Institute Maastricht (CARIM), Maastricht University, 6200 MD Maastricht, The Netherlands; m.ploeg@maastrichtuniversity.nl (M.C.P.); c.munts@maastrichtuniversity.nl (C.M.); frits.prinzen@maastrichtuniversity.nl (F.W.P.); marc.vanbilsen@maastrichtuniversity.nl (M.v.B.); 2 Discovery and Translational Science Department, Leeds Institute of Cardiovascular and Metabolic Medicine, School of Medicine, University of Leeds, Leeds LS2 9JT, UK; n.a.turner@leeds.ac.uk; 3 Multidisciplinary Cardiovascular Research Centre, University of Leeds, Leeds LS2 9JT, UK

**Keywords:** mechanosensing, cardiac fibroblast, brain natriuretic peptide, piezo1, stretch

## Abstract

In response to stretch, cardiac tissue produces natriuretic peptides, which have been suggested to have beneficial effects in heart failure patients. In the present study, we explored the mechanism of stretch-induced brain natriuretic peptide (Nppb) expression in cardiac fibroblasts. Primary adult rat cardiac fibroblasts subjected to 4 h or 24 h of cyclic stretch (10% 1 Hz) showed a 6.6-fold or 3.2-fold (*p* < 0.05) increased mRNA expression of Nppb, as well as induction of genes related to myofibroblast differentiation. Moreover, BNP protein secretion was upregulated 5.3-fold in stretched cardiac fibroblasts. Recombinant BNP inhibited TGFβ1-induced Acta2 expression. Nppb expression was >20-fold higher in cardiomyocytes than in cardiac fibroblasts, indicating that cardiac fibroblasts were not the main source of Nppb in the healthy heart. Yoda1, an agonist of the Piezo1 mechanosensitive ion channel, increased Nppb expression 2.1-fold (*p* < 0.05) and significantly induced other extracellular matrix (ECM) remodeling genes. Silencing of Piezo1 reduced the stretch-induced Nppb and Tgfb1 expression in cardiac fibroblasts. In conclusion, our study identifies Piezo1 as mediator of stretch-induced Nppb expression, as well as other remodeling genes, in cardiac fibroblasts.

## 1. Introduction

Mechanical factors influence the form and function of cells [1,2,3,4]. Specifically in the heart, mechanical signals include the forces of cyclic contraction and relaxation of the myocardial walls and the hemodynamic load leading to stretch of the cardiac chambers during the filling phase, and increased wall stress during the contraction phase [5]. These factors are known to regulate myocardial function, gene expression and structural appearance [6,7].

Cardiovascular tissue is composed of cardiomyocytes, fibroblasts, vascular and immune cells which reside within the myocardial extracellular matrix (ECM) [8]. The ECM provides structure, transmits mechanical forces and modulates cell function [8,9]. Cardiac fibroblasts play an important role in the regulation of the ECM, by synthesizing structural ECM proteins (i.e., collagens), ECM degrading matrix metalloproteinases (MMPs and TIMPs) [10], growth factors such as transforming growth factor β1 (TGFβ1) and matricellular proteins like tenascin C (TNC) [11] and connective tissue growth factor (CTGF) [12]. Matricellular proteins gain increasing attention for their significant role in cardiac remodeling [11]. In response to injury, cardiac fibroblasts become activated and differentiate to so-called myofibroblasts [10,13]. These myofibroblasts have special morphological and functional characteristics, such as the expression of alpha smooth muscle actin (aSMA, encoded by ACTA2 gene) [10,14]. TGFβ1 is known as established stimulus for myofibroblast differentiation [15,16]. Even though myofibroblast differentiation is an essential process in normal wound healing, it can result in pathological fibrosis in cases of prolonged injury or loss of regulatory mechanisms [8]. In addition to biochemical factors, mechanical cues such as mechanical strain and ECM stiffness also play an important role in regulating myofibroblast differentiation [17,18]. Cardiac fibroblasts express a number of different mechanosensitive ions channels that are coupled to alteration of cellular phenotype and function [19].

Cardiovascular tissues can produce natriuretic peptides in response to wall stretch [20,21,22,23,24]. There are three types of natriuretic peptides: atrial natriuretic peptide (ANP, encoded by NPPA gene), brain natriuretic peptide (BNP, encoded by NPPB gene), and C-type natriuretic peptide (CNP, encoded by NPPC gene) [25,26]. ANP and BNP are found in multiple tissues, but they are produced primarily in the cardiac atria or ventricles, respectively [27,28,29,30]. CNP is mainly produced in the endothelium [27,31]. Cyclic stretch induced increased Nppa and Nppb expression in adult rabbit cardiomyocytes [32] and human embryonic stem cell–derived cardiomyocytes (hESC-CMs) [33].

BNP inhibits collagen production and fibroblast proliferation [10], and the TGFβ-activation of pro-fibrotic and inflammatory genes in cultured human cardiac fibroblasts [34]. Nppb knock out mice subjected to pressure overload by aortic constriction show increased fibrosis as well as increased mRNA levels of Tgfb3 and Col1a1 [35]. The beneficial effects of BNP in the heart has led to pharmacotherapy aimed at increasing BNP signaling in heart failure patients [36,37].

While it is generally accepted that cardiomyocytes produce BNP [32,38,39,40,41,42], some studies show that it is also synthesized by cardiac fibroblasts [10,24,38,43]. A recent paper found that stretch of human cardiac fibroblasts increased NPPB expression [43]. However, the mechanism of stretch-induced NPPB expression in cardiac fibroblasts is unknown. Blythe and colleagues very recently identified the presence of Piezo1 as a functional Ca^2+^-permeable mechanosensitive ion channel in both murine and human cardiac fibroblasts [44]. Therefore, we hypothesized that stretch-induced NPPB expression by cardiac fibroblasts is mediated by the mechanosensitive ion channel Piezo1.

## 2. Materials and Methods

### 2.1. Isolation of Cardiac Fibroblasts and Cardiomyocytes

Cardiac fibroblasts were isolated from cardiac ventricles (combined left and right) of adult surplus rats (*n* = 31) from any age, weight, sex or breed. Most of the rats used were either from the Lewis or Wistar strain and aged between 5 and 52 weeks. Rat cardiac ventricular fibroblasts were isolated and cultured as previously described [9,45,46] in Dulbecco’s modified eagles medium (DMEM; no. 22320, Gibco, Invitrogen, Breda, the Netherlands) supplemented with 10% (*v*/*v*) fetal bovine serum (FBS, Gibco), gentamicin (50 µg/mL, Gibco), 1% (*v*/*v*) Insulin-Transferrin-Selenium-Sodium Pyruvate (ITS-A, Gibco) and basic fibroblast growth factor (1 ng/mL, Gibco) (“CF culture medium”). The vast majority of these cells are fibroblast-like cells and these primary fibroblasts were used between passage 1–3. Cardiomyocytes were isolated from the left ventricle of adult male Sprague Dawley rats (*n* = 6 age 10–20 weeks) essentially as described previously [32,47]. Experiments were performed with approval of the Animal Ethical Committee of Maastricht University (DEC-2007-116, July 31, 2007) and conform to the national legislation for the protection of animals used for scientific purposes.

### 2.2. Experimental Stretch Protocols

Cardiac fibroblasts (10,000 cells/cm^2^) were plated on bioflex plates (6-well Bioflex plates, precoated with collagen-I, Flexcell Dunn Labortechnik, Asbach, Germany) in CF culture medium. The next day, CF culture medium was replaced by DMEM supplemented with gentamicin (50 µg/mL, Gibco). After 24 h, cardiac fibroblasts were subjected to 10% cyclic (1 Hz) equibiaxial stretch, (Flexcell FX-4000 strain unit, Dunn Labortechnik) for 4 h, 6 h or 24 h. Control, non-stretched cells were subjected to identical conditions however, without stretch being applied.

### 2.3. Experimental Stimuli

To determine regulation and effects of BNP, cardiac fibroblasts (10,000 cells/cm^2^) were serum-starved for 24 h before incubation with TGFβ1 (1 ng/mL, R&D systems, Minneapolis, MN, USA), Yoda1 (10 µM, Tocris, Bristol, UK) and BNP (1 µM, R&D systems) for 4 h or 24 h.

### 2.4. Gene Expression Analysis

Total RNA was isolated from cells using an RNA isolation kit (Omega Biotek, Norcross, GA, USA) and reversed transcribed into cDNA using the iScript cDNA synthesis kit (Biorad, Hercules, CA, USA) according to the manufacturer’s instructions. Real-time PCR was performed on an iCycler accompanied by the My IQ single color real-time PCR detection system using iQ SYBR-Green Supermix (Biorad) [9]. Gene expression levels of Alpha-smooth muscle actin (Acta2), Connective tissue growth factor (Ctgf), Transforming growth factor beta 1 (Tgfb1), Tenascin C (Tnc), Piezo1, Atrial Natriuretic peptide (Nppa), C-Type natriuretic peptide (Nppc) and Brain Natriuretic Peptide (Nppb) were normalized using the housekeeping gene Cyclophilin-A (Cyclo), and their relative expression was calculated using the comparative threshold cycle (Ct) method by calculating 2^ΔCt^ (e.g., 2^(Cyclophilin Ct –BNP Ct)^). The gene expression values were multiplied by 1000 (formula 1000 * 2^ΔCt^), to enhance readability. The sequences of the specific primers used are provided below (Table 1).

### 2.5. BNP ELISA

Conditioned media were collected after the 24 h stretch-experiments and stored at −80 °C for subsequent analysis. The conditioned media were concentrated (approximately 10-fold) using Amicon Ultra 3k devices (Merck-Millipore, Burlington, MA, USA) and the concentration of BNP was determined by ELISA (ab108815, Abcam, Cambridge, UK) according to the manufacturer’s instructions.

### 2.6. Gene Silencing

Cardiac fibroblasts (10,000 cells/cm^2^) were plated on bioflex plates (6-well Bioflex plates, precoated with collagen-I, Flexcell Dunn Labortechnik) in CF culture medium and transfected with 10 nM Piezo1-specific Silencer Select Pre-Designed siRNA (4390771, siRNA s107968, Life Technologies, Carlsbad, CA, USA) or Silencer Select Negative Control No. 1 siRNA (4390843, Life Technologies) using Lipofectamine RNAiMAX reagent (Life Technologies) in Opti-MEM (Gibco) according to the manufacturer’s instructions. After 72 h cells were exposed to the stretch protocol described above.

### 2.7. Statistics

Data are presented as mean or individual data points and were analyzed with Wilcoxon matched pairs test or Friedman test, with Dunn posthoc test where appropriate. Differences were considered statistically significant when *p* < 0.05.

## 3. Results

### 3.1. Mechanical Stretch Induces BNP Expression in Cardiac Fibroblasts

**Figure 1:**
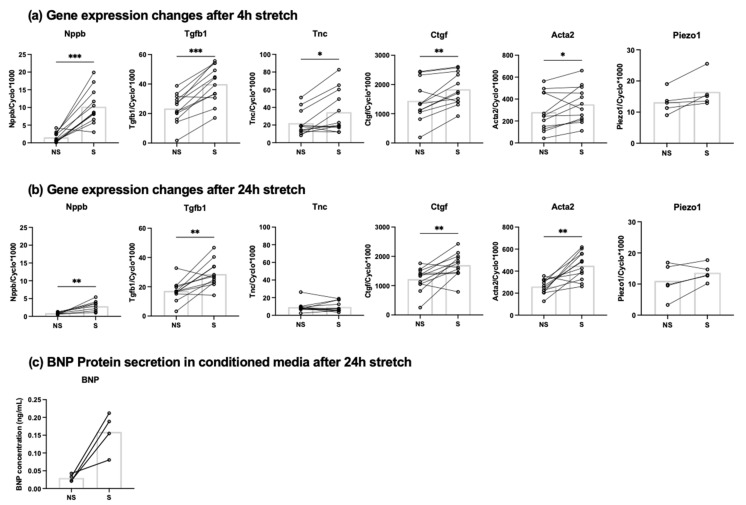
Relative mRNA expression levels of Nppb, Tgfb1, Tnc, Ctgf, Acta2, and Piezo1 in cardiac fibroblasts exposed to 10% 1 Hz cyclic stretch (S) or non-stretched (NS) conditions for 4 h (*n* = 5–12) (**a**) or 24 h (*n* = 5–12) (**b**). BNP-protein concentration in conditioned media from stretched and non-stretched cardiac fibroblasts (*n* = 4) (**c**). * *p* < 0.05; ** *p* < 0.01; *** *p* < 0.001. Bar indicates mean.

Cardiac fibroblasts exposed to cyclic stretch (10%, 1 Hz) for 4 h showed a significant increase in mRNA expression of Tgfb1, Tnc, Ctgf and Acta2 compared to non-stretched controls (Figure 1a). After 24 h of cyclic stretch the effect remained for Tgfb1, Ctgf and Acta2 (Figure 1b). Interestingly, Nppb mRNA expression was significantly upregulated by 6.6-fold after 4 h stretch (Figure 1a) and 3.2-fold after 24 h stretch (Figure 1b) compared to non-stretched controls. BNP-protein secretion was upregulated by 5.3-fold in stretched cells compared to non-stretched cells, measured from conditioned media after 24 h stretch (Figure 1c). mRNA expression of Nppa and Nppc were below the detection limit. Cyclic stretch did not influence the mRNA expression of the mechanosenstive ion channel Piezo1 after 4 h (Figure 1a) or 24 h (Figure 1b)

### 3.2. Recombinant BNP Inhibits Profibrotic Gene Expression in Cardiac Fibroblasts

**Figure 2:**
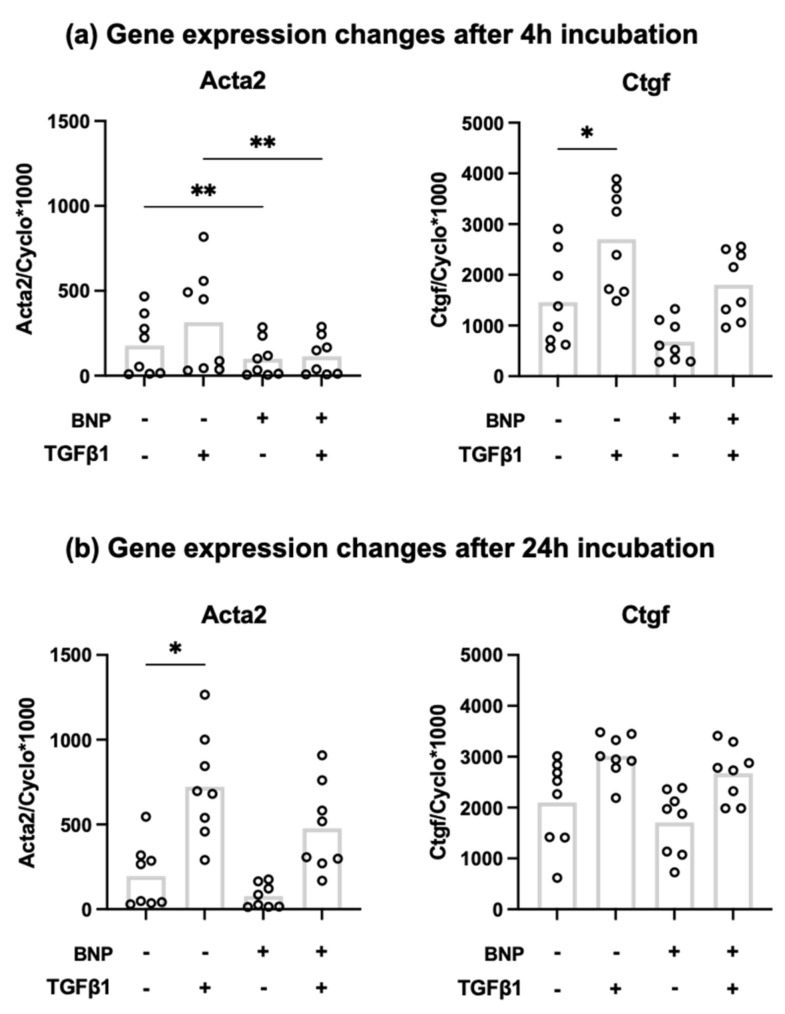
Relative mRNA expression levels of Ctgf and Acta2 in cardiac fibroblasts exposed to recombinant BNP and/or TGFβ1 after 4 h (**a**) and 24 h (**b**) (*n* = 8) * *p* < 0.05; ** *p* < 0.01. Bar indicates mean.

To confirm the anti-fibrotic effect of BNP [34], cardiac fibroblasts were stimulated with recombinant BNP with or without TGFβ1 for 4 h or 24 h. The expression of Acta2 and Ctgf in cardiac fibroblasts was investigated by RT-qPCR. Acta2 showed a significant reduction in expression after the addition of BNP, with and without TGFβ1 after 4 h (Figure 2a). This effect was not maintained after 24 h (Figure 2b). A similar trend was observed for Ctgf, but this failed to reach statistical significance.

### 3.3. Both Cardiomyocytes and Cardiac Fibroblasts Express Nppb

**Figure 3:**
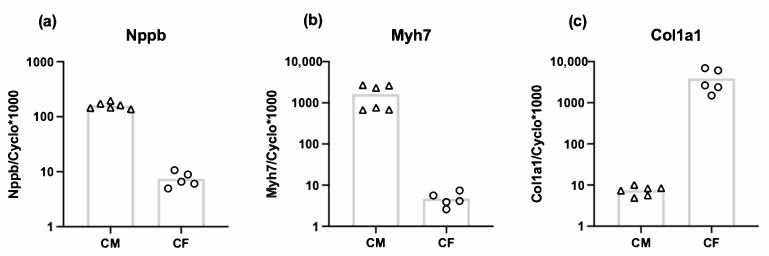
Relative mRNA expression levels of Nppb (**a**) Myh7 (**b**) and Col1a1 (**c**) in cardiomyocytes (CM) (*n* = 6) and cardiac fibroblasts (CF) (*n* = 5) (presented on a logarithmic scale). Bar indicates mean.

To investigate the relative mRNA expression of Nppb by cardiac fibroblasts compared to cardiomyocytes, we performed RT-qPCR on adult rat cardiomyocytes and cardiac fibroblasts. Cardiac fibroblasts showed 300-fold lower expression of Myh7 (myocyte marker) (Figure 3b) and 500-fold higher expression of Col1a1 compared to cardiomyocytes (Figure 3c). The Nppb expression in cardiomyocytes was 20-fold higher compared to cardiac fibroblasts, indicating that fibroblasts are not the main source of myocardial Nppb expression, at least under basal conditions (Figure 3a).

### 3.4. Stretch-Induced Nppb and Tgfb1 Expression Are Mediated by Piezo1

**Figure 4:**
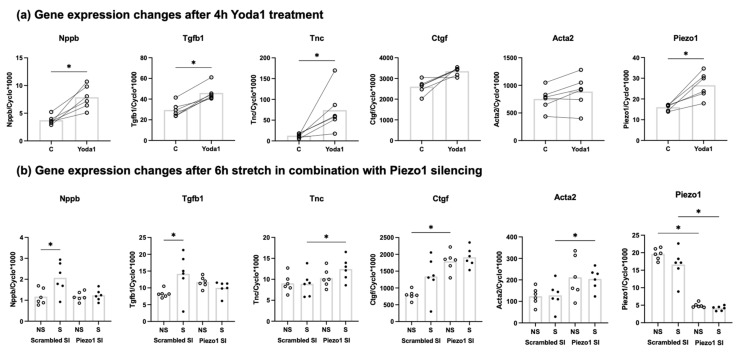
Relative mRNA expression levels of Nppb, Tgfb1, Tnc, Ctgf, Acta2, and Piezo1 in cardiac fibroblasts after stimulation with Piezo1 agonist Yoda1 after 4 h (*n* = 6) (**a**) Relative mRNA expression of Nppb, Tgfb1, Tnc, Ctgf, Acta2, and Piezo1 in cardiac fibroblasts exposed to 10% 1 Hz cyclic stretch for 6 h (S) or non-stretched (NS) conditions after transfection with either control siRNA (Scrambled SI) or Piezo1-specific siRNA (Piezo1 SI) (*n* = 6) (**b**) * *p* < 0.05. Bar indicates mean.

To gain insight into the functional role of Piezo1 activation in cardiac fibroblasts, we first investigated the effect of the Piezo1 agonist Yoda1. Treatment of unstretched fibroblasts with 10 µM Yoda1 for 4 h significantly increased mRNA expression of Tgfb1, Tnc and Nppb (Figure 4a). Yoda1 stimulation also gave a significant increased expression of Piezo1 mRNA.

Next, the effect of siRNA-mediated Piezo1 silencing was explored. Piezo1 silencing was successful in reducing the expression of Piezo1 by approximately 80% (Figure 4b). Piezo1 silencing increased the expression of Ctgf under non-stretched conditions, and the Tnc and Acta2 expression levels under stretch. The stretch-induced increase in Tgfb1 expression was absent following Piezo1-silencing (Figure 4b). Piezo1 silencing also prevented stretch-induced Nppb expression, supporting the hypothesis that stretched induced Nppb expression is Piezo1 mediated.

## 4. Discussion

The present study identifies Piezo1 as the mechanosensitive ion channel responsible for the stretch-induced Nppb and Tgfb1 expression in cardiac fibroblasts. Our study also confirms the finding of Watson and colleagues of stretch-induced Nppb upregulation in human ventricular cardiac fibroblasts [43] and the anti-fibrotic effect of BNP [10,34].

Natriuretic peptides are thought to be part of a potentially beneficial counter-regulatory system in heart failure [48]. BNP might prevent the development of cardiac fibrosis by serving as a cardiomyocyte-derived antifibrotic signal to cardiac fibroblasts during the process of cardiac remodeling [35]. However, others have shown Nppb expression by fibroblasts [10,38,49]. We confirmed the expression of Nppb in cardiac fibroblasts, on mRNA as well as protein level in response to cyclic stretch. Although expression levels of Nppb in our isolated cardiac fibroblasts were lower than found in isolated cardiomyocytes, it is important to note that the cells were derived from healthy animals. Furthermore, the locally produced BNP by cardiac fibroblasts might still have important autocrine or paracrine functions. Cyclic stretch also upregulated the mRNA expression of Tgfb1, Tnc, Ctgf and Acta2 after 4 h, which was maintained for all but Tnc after 24 h. Together, these genes are all related to myofibroblast differentiation and cardiac remodeling [10,11,12,14,15,16]. Their upregulation gives some implications on the process of myofibroblast differentiation being started or already ongoing. Cyclic stretch did not affect the mRNA expression of Piezo1, after 4 h or 24 h.

The antifibrotic effect of BNP has been shown previously [34], which we confirmed specifically for myofibroblast differentiation related gene Acta2. Therefore, it is possible that BNP produced by fibroblasts acts as a local autocrine/paracrine factor modulating cardiac fibroblast activation and tissue remodeling within the heart [50]. Differences between 4 h and 24 h incubation with BNP and/or TGFβ1 in our cell culture experiments might be due to the relative short half-life of both BNP and TGFβ1. Therefore, both products are degraded within hours and their effects might be stronger at 4 h as compared to 24 h.

The well-known Piezo1 agonist Yoda1 increased the mRNA expression on Nppb, Tgfb, Tnc and Piezo1. For Nppb, Tgfb1 and Tnc this is in accordance with the results of 4 h and 24 h of cyclic stretch, activating the Piezo1 mechanosensitive ion channel. The effect of Yoda1 on Piezo1 expression levels is unexpected as previous experiments showed no effect of 24 h Yoda1 stimulation on Piezo1 expression levels in murine cardiac fibroblasts [44]. The induction of Piezo1 expression by its agonist Yoda1 implies a positive feedback loop, which merits further investigation. In contrast to Yoda1, cyclic stretch did not affect Piezo1 mRNA expression. A possible explanation for the difference between the cyclic stretch and Yoda1, might be that Yoda1 is a stronger stimulus than stretch. On the other hand, stretch significantly increased the mRNA expression of Acta2 and Ctgf after 4 h, but Yoda1 does not. This might be due to the low numbers of cardiac fibroblast isolations (*n* = 6) used in the Yoda1 experiment; Although no statistically significant difference was found, Yoda1 increased both Ctgf (*p* = 0.06) and Acta2 (*p* = 0.1) mRNA levels, which fits with the results from the stretch experiments.

Silencing Piezo1 increased the mRNA expression levels of Ctgf in non-stretched cells and of Tnc and Acta2 in stretched cells. It is possible that the Piezo1 channel is active under control conditions, and inhibits the expression of these genes. However, this would contradict our observation that Yoda1 (which activates Piezo1) stimulated Tnc expression. Possibly, silencing of Piezo1 led to cellular compensatory mechanisms that indirectly affected the expression of these genes. Obviously, the mechanisms by which Piezo1-silencing affected Ctgf, Tnc and Acta2 mRNA expression levels are not clear and further studies are warranted to investigate this. Previously, our colleagues from the Leeds group showed that IL-6 expression and secretion in cardiac fibroblasts was inhibited by Piezo1 silencing, indicating a role for Piezo1 signaling in the expression of this pro-inflammatory gene [44]. Interestingly, this was depending on substrate stiffness and/or composition as the effect was absent when cells were cultured on regular non-coated cell culture plates (rigid plastic) [18,44]. Stiffness of standard plastic culture plates are estimated in the gigapascal (GPa) range [51]. The Bioflex plates we have used for the stretch experiment have a stiffness 1000 times less (~1 megapascal, MPa) than those standard culture plates. However, the stiffness of healthy myocardial tissue is estimated to be 10 kilopascal (kPa) [51,52,53], making myocardial tissue stiffness 100 times less than the Bioflex plates. Therefore, even though the Bioflex plates are softer than regular culture plates, they are still stiff compared to healthy myocardial tissue. Of note, the stiffness of fibrotic myocardium is estimated at 20–100 kPa [51,52,53]. It has also been shown previously that Piezo1 reacts to stiffness, in both stem cells [54] and atrial fibroblasts [55].

In the present study, we did not investigate the mechanism of Piezo1-induced effects on gene expression. However, the Leeds group has reported on how Il-6 expression is linked to Piezo1 [44]. They suggest an important role for the p38 MAPK pathway in Piezo1-induced Il-6 gene expression, in which p38 activation was depending on extracellular Ca^2+^. A similar activation pathway might be the case for Nppb or Tgfb1, but the mechanism of Piezo1 activation on Nppb or Tgfb1 expression requires further research.

In conclusion, the present study shows that both stretch-induced Nppb and Tgfb1 expression in adult rat cardiac fibroblasts is mediated by the mechanosensitive ion channel Piezo1. Furthermore, BNP protein levels were upregulated in stretched cardiac fibroblasts and recombinant BNP inhibited TGFβ1-induced Acta2 expression in cardiac fibroblasts. Together, these results indicate that Piezo1 is an important mechanosensitive ion channel mediating stretch-induced activation of cardiac fibroblasts.

## Figures and Tables

**Table 1:** Gene-specific primer sequences used for quantitative real-time PCR.

Gene	Forward Primer	Reverse Primer
Alpha-smooth muscle actin (Acta2)	AAGGCCAACCGGGAGAAAAT	AGTCCAGCACAATACCAGTTGT
Connective tissue growth factor (Ctgf)	CACAGAGTGGAGCGCCTGTTC	GATGCACTTTTTGCCCTTCTTAATG
Transforming growth factor, beta 1 (Tgfb1)	GCACCATCCATGACATGAAC	GCTGAAGCAGTAGTTGGTATC
Tenascin C (Tnc)	TCTGTCCTGGACTGCTGATG	TGGCCTCTCTGAGACCTGTT
Piezo1	TTGCGTACGTTCACGAAGGA	TTCGCTCACGTAAAGCTGGT
Atrial Natriuretic peptide (Nppa)	ATCACCAAGGGCTTCTTCCT	TGTTGGACACCGCACTGTAT
Brain Natriuretic Peptide (Nppb)	AGACAGCTCTCAAAGGACCA	CTATCTTCTGCCCAAAGCAG
C-Type natriuretic peptide (Nppc)	ACAAAGGCGGCAACAAGAAG	GCAGTTCCCAATCCGCCG
Cyclophilin-A (Cyclo)	CAAATGCTGGACCAAACACAA	TTCACCTTCCCAAAGACCACAT

## Data Availability

The data presented in this study are available from the corresponding author upon request.
